# Oral medicinal cannabinoids to relieve symptom burden in the palliative care of patients with advanced cancer: a double-blind, placebo controlled, randomised clinical trial of efficacy and safety of cannabidiol (CBD)

**DOI:** 10.1186/s12904-019-0494-6

**Published:** 2019-12-06

**Authors:** Phillip Good, Alison Haywood, Gauri Gogna, Jennifer Martin, Patsy Yates, Ristan Greer, Janet Hardy

**Affiliations:** 1 Mater Health Services, Mater Research Institute-University of Queensland, St Vincent’s Private Hospital Brisbane, Brisbane, Qld Australia; 2 School of Pharmacy and Pharmacology, Menzies Health Institute Queensland, Griffith University, Gold Coast, Australia; 3 Mater Research Institute – The University of Queensland, Brisbane, Australia; 4 Greenslopes Private Hospital, Gold Coast Health Service, Greenslopes, Australia; 5 The Australian Centre for Cannabinoid Clinical and Research Excellence (ACRE), New Lambton Heights, Australia; 6 Discipline of Clinical Pharmacology, School of Medicine and Public Health, University of Newcastle, Callaghan, Australia; 7 School of Nursing, Queensland University of Technology, Victoria Park Rd, Kelvin Grove, QLD 4059 Australia; 8 Centre for Palliative Care Research and Education, Queensland Health, Brisbane, Australia; 9 Torus Research, Mater Research Institute-University of Queensland, QLD, St Lucia, Australia; 10 Mater Health Services, Mater Research Institute-University of Queensland, Brisbane, Queensland Australia

**Keywords:** Cannabis, Cannabidiol, Cancer, Symptom control, RCT, Palliative care

## Abstract

**Background:**

Despite improvements in medical care, patients with advanced cancer still experience substantial symptom distress. There is increasing interest in the use of medicinal cannabinoids, but there is little high quality evidence to guide clinicians. This study aims to define the role of cannabidiol (CBD) in the management of symptom burden in patients with advanced cancer undergoing standard palliative care.

**Methods and design:**

This study is a multicentre, randomised, placebo controlled, two arm, parallel trial of escalating doses of oral CBD. It will compare efficacy and safety outcomes of a titrated dose of CBD (100 mg/mL formulation, dose range 50 mg to 600 mg per day) against placebo. There is a 2-week patient determined titration phase, using escalating doses of CBD or placebo to reach a dose that achieves symptom relief with tolerable side effects. This is then followed by a further 2-week assessment period on the stable dose determined in collaboration with clinicians.

**Discussion:**

A major strength of this study is that it will target symptom burden as a whole, rather than just individual symptoms, in an attempt to describe the general improvement in wellbeing previously reported by some patients in open label, non controlled trials of medicinal cannabis. Randomisation with placebo is essential because of the well-documented over reporting of benefit in uncontrolled trials and high placebo response rates in cancer pain trials. This will be the first placebo controlled clinical trial to evaluate rigorously the efficacy, safety and acceptability of CBD for symptom relief in advanced cancer patients. This study will provide the medical community with evidence to present to patients wishing to access medicinal cannabis for their cancer related symptoms.

**Trial registration number:**

ALCTRN12618001220257 Registered 20/07/2018.

## Background

Despite advances in medicine, patients with advanced cancer can still experience substantial symptom burden that causes distress [1]. Palliative care aims to provide a patient-centred, holistic approach to improve patients’ health and well-being. There is a wide range of medications available, but the control of many symptoms (e.g. pain, nausea, anorexia, anxiety,) remains an ongoing challenge [2].

There is increasing interest in the use of medicinal cannabinoids for the relief of symptoms in palliative care patients [3]. There has been changes in legislation in Australia (both federally and in states) that has now provided pathways to prescribe medicinal cannabinoids for a range of indications for a number of indications including – palliative care, intractable epilepsy, chronic pain, spasticity associated with multiple sclerosis, and chemotherapy induced nausea and vomiting (CINV) [4].

Cannabis contains more than 500 bioactive compounds, including more than seventy different cannabinoids [5]. The main cannabinoids are delta-9-tetrahydrocannabinol (THC) and cannabidiol (CBD). THC is thought to be the main psychoactive component of cannabinoids Potential benefits of THC can include pain control,, nausea improvement, and relaxation of muscles with potential side effects including psychosis, sedation and intoxication. The recommended dose range for oral THC varies from 2.5 to 40 mg/day [6]. CBD is not intoxicating, and in animals has demonstrated some benefits in anxiety, psychosis, inflammation, epilepsy and demonstrated neuroprotective effects [7]. CBD is also considered to mediate many of the adverse psychotropic effects of THC, although this research is still emerging [8]. CBD has been used with a dosing in the range of 40 to 1280 mg/day orally [9].

There is little high quality evidence of benefit to date for the use of medicinal cannabinoids. The most recent review from the USA National Academies of Sciences, Engineering, and Medicine found evidence for the use of medicinal cannabinoids for treatment of some types of chronic pain, CINV and spasticity in multiple sclerosis in some patients, with moderate evidence for sleep disorders [10]. Cannabinoid products are licensed for a range of conditions in different countries, with little consistency between countries for indication and dosing [11]. Whilst cannabinoids may have potential clinical benefits, their use is not without possible adverse effects and further research is needed define their role in medical practice [3, 11].

There are many unknowns when it comes to prescribing medicinal cannabis [11]. This includes the formulation of the product, the ratios of THC or CBD, dosing levels and the best route of delivery. Ongoing concerns remain around the uncertainty over toxicity and abuse potential. The 1:1 THC/CBD ratio used in formulations may deliver sufficient CBD to ameliorate the psychotoxic effects of THC, but is unlikely to produce significant CBD therapeutic effects.

We hypothesize that a higher CBD dose is required in order to deliver therapeutic effects. However, to date there has been no formal examination of the effect of different dose of CBD. We will therefore compare efficacy and safety outcomes of a dose escalated 100 mg/mL formulation against placebo, with CBD escalated to doses previously shown to be safe.

## Methods

### Aims and objectives

This study aims to define the role of CBD in the management of symptoms in patients with advanced cancer undergoing standard palliative care. The hypothesis is that medicinal cannabinoids will reduce the total symptom burden in these patients compared to placebo.

The primary objective is to assess the effect of escalating doses of a CBD against placebo on total symptom scores at day 14, as measured by the Edmonton Symptom Assessment Scale (ESAS) [12]. Secondary objectives are to establish a patient determined effective dose range of the CBD, to assess the effect on symptom scores at days 7, 21 and 28, the change in total physical and emotional sores, global impression of change, anxiety and depression, opioid use, quality of life, and to document adverse effects associated with CBD use at different dosages.

### Study design

This study is a multicentre, randomised, placebo controlled, two arm parallel trial of escalating doses of oral CBD (100 mg/ml oil formulation). There is a 2-week patient determined titration phase, using escalating doses of CBD or placebo, to reach a dose that achieves symptom relief with tolerable side effects. This is then followed by a further 2-week assessment period on the stable dose determined in collaboration with clinicians.

Eligible patients will be randomly assigned in equal numbers to one of the two study arms. Randomisation schedules will be developed for each site using random number tables, computer generated at an independent centre. Treatment for each patient will be allocated according to a block randomisation schedule held by the central registry. Block randomisation within each centre will ensure uniform allocation to each arm in each site. Pharmacy will be notified of a participant, and a completed script with the participant’s study ID number will be given to the site pharmacy. The pharmacist will randomise the participant according to the schedule and dispense the medication in a labelled bottle. The participant ID, allocation number, date of request, preparation and dispensing will be recorded in a log maintained by the site pharmacist for each randomisation.

All participants, caregivers, investigators and clinical staff will remain blind to study assignment until trial completion. The code will only be broken in cases of a clinical emergency. An investigator not directly involved in the randomisation of patients will keep a blinded copy of the randomisation schedule and will be contacted in the event of the need to un-blind. All study drugs and placebo will be in oil solution form and identical in appearance. All oil solutions will be matched for taste, colour and bottle size to preserve the blinding irrespective of the contents, each participant will receive the oil solution in a prepacked bottle labelled with their individual trial participation ID number, and consecutively numbered according to the randomisation scale.

### Intervention

Arm 1 of the trial consists of CBD 100 mg oral oil solution (GD Pharma Pty Ltd) and Arm 2 a matching placebo oral oil solution. Participants are asked to take daily doses as per dosing schedule instructions. The study drug is to commence on the day of baseline assessment, continuing for a maximum of 28 days.

The cannabinoid oils and placebo will be dispensed in identical 25 mL bottles containing a sufficient quantity of oil. Participants will be given replacement bottles to complete the study as required. Participants will be educated on how to administer daily doses using supplied 1 mL syringes appropriate for the dose. Cannabinoid oil that has been allocated to a participant and not used will be returned to the research nurse/research officer and destroyed by local pharmacy as per pharmacy guidelines.

Dose titration will be confirmed following regular consultation. Each participant will be advised to increase their dose according to a schedule until they are satisfied with their symptom improvement or they experience unacceptable side effects. Dose titration downwards will also be allowed, in consultation with research staff. The participant in collaboration with research staff will define the dose level at which they will continue until the primary outcome point of 14 days. Participants will then be given the option of remaining on the blinded oil solution for a further 2 weeks (28 day total) for continuing assessment of efficacy and adverse events. Assessment at 28 days is a secondary end-point.

Participants will be recruited from five sites of the Queensland Palliative Care Research Group (QPCRG). It is anticipated that this will be predominately an outpatient study.

All participants will be given standard palliative care according to the local practice of the recruiting centre [12]. They will continue all current medications including opioids, antiemetics, sedatives and specific anti-cancer therapy (including chemotherapy, immunotherapy, and radiotherapy).

### Study participants

Patients who meet the inclusion criteria will have an advanced histologically proven cancer diagnosis (metastatic or locally advanced), be known to and be receiving palliative care at the recruiting centre, have an ESAS Total Symptom Distress Score (TSDS) of ≥10/90 for cancer-related symptoms, and at least one individual ESAS score ≥ 3/10 [13]. They must have a Performance Status AKPS (Australia-modified Karnofsky Scale score) of ≥30/100 [14], be aged ≥25 years, have a negative THC urine test at commencement of trial, be able to tolerate oral medications and must be either English-speaking or have an interpreter available.

Participants must have a negative pregnancy urine test at eligibility (only if of reproductive potential) and agree to avoid pregnancy during the study and 12 weeks following the last dose of the study drug. Males must agree to avoid fathering a child and to not donate sperm during the study and for at least 12 weeks following the last dose of the study drug. Further to this the Alcohol, Smoking and Substance Involvement Screening Test (ASSIST) [15] will be conducted to determine eligibility. It is designed to determine whether harmful substance are being used and go undetected or become worse. The assessment is comprised of 8 questions assessing, tobacco, alcohol, cocaine, amphetamine-type stimulants, sedatives, hallucinogens, opiates and ‘other drugs’.

Exclusion criteria will include a history of hypersensitivity to any cannabinoid, unstable untreated cardiovascular disease, severe hepatic impairment (total bilirubin ≥1.5 times the upper limit of the normal range, aspartate aminotransferase (AST), and alanine aminotransferase (ALT) ≥3.0 times the upper limit of the normal range; subjects with liver metastasis may have an AST and ALT of ≥5.0 times the upper limit of normal), severe renal impairment (eGFR ≤20 mL/min/1.73 m2), a history of psychiatric disorders (severe depression or anxiety, personality disorder, psychosis, schizophrenia, first degree relative with schizophrenia and/or suicidal ideation), cognitive impairment (SLUMS - St Louis University Mental Status examination ≤20/30) [16] and a known substance use disorder (ASSIST - Alcohol, Smoking and Substance Involvement Screening Test) examination scoring > 27 for any substance).

Potential participants will be excluded if they have a history suggesting that drug diversion may be a risk for them or their family/carers, have participated in a trial of a new clinical entity within the last 28 days, or treatment with a new specific anticancer agent (chemotherapy, targeted or hormonal therapy) or radiation within the previous 7 days.

### Consent process

The process for obtaining consent for this study will be exchanging information between the study staff and potential participants and any other person the participant wishes to include in the discussion. A participant information sheet (PICF) will be provided in written form and will be used as the basis for the discussion. This will cover the purpose, expected procedures, participant requirements, risks, benefits, burdens and side effects that are expected or possible during the study. Potential participants will be given the opportunity (in time and physical capacity) to consider the study, formulate any questions. All questions will be addressed and answered fully. An actual time point for consent will not be specified as this will be determined by the person’s physical condition. The consent form is to be completed by trained study team members in accordance with the requirements of the approving ethics committee. The form is to be signed and dated by the participant.

### Assessments

Participants will receive 2 times/weekly research nurse phone calls in the first 2 weeks and out-patient clinic medical review visits at days 7, 14, 21 day 28, with outcome measures at these points.

Symptom burden will be measured using the Edmonton Symptom Assessment Scale (ESAS). Confirmed documented disease status will be assessed at days 14 and 28, where applicable.

A routine haematology and biochemistry screen including liver function tests will be taken at eligibility/baseline assessment. Blood for C-reactive protein (CRP) as a basic test of inflammation will be taken at baseline, day 14 and day 28. All consenting participants will have a urine test to exclude recent use of THC related products as a pre screen.. At day 14 a urine sample will be collected and stored (frozen) until the completion and un-blinding of the study. Samples will be stored on site and transported to central storage in batches over the course of the study. These samples will be for post-trial analysis for evidence of no THC product use during the trial.

Participants will be contacted at day 56 (+ 4 weeks post last dose). Date of death will be recorded for all participants up the census point. The study schedule can be viewed in Table 1.

**Table 1:**
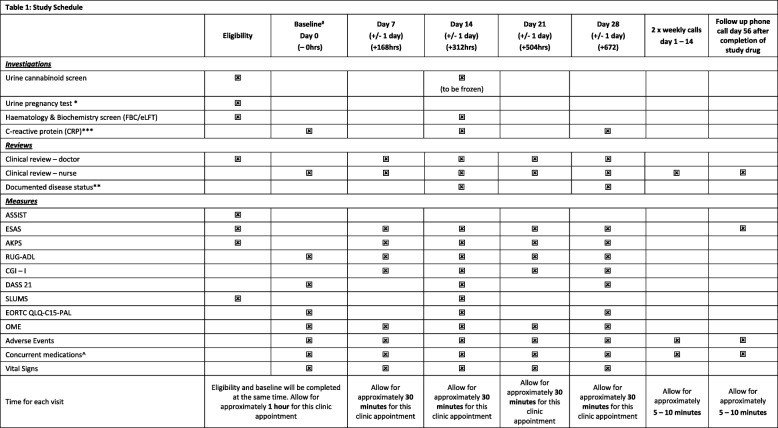
Study schedule

*only those women of reproductive potential

**Confirm documented disease status (CR, PR, SD, PD or ND)

***optional or if blood being taken as part of routine care

^ including cannabis and complimentary medicine use

#eligibility data to be used as baseline as long as within 3 days of each oth

### Outcomes and assessment tools

The primary outcome is a change from baseline of total ESAS TSDS at day 14. Clinically significant change is determined to be an improvement of the TSDS of ≥6.

The ESAS is a 9 item inventory rated on a 11 point scale anchored at 1 (no problem) to 10 (worst problem). It assesses both physical and psychological symptoms, plus general wellbeing. It has been validated in the assessment of symptoms in cancer patients [13].

Secondary Outcomes include:

1. Patient determined effective dose of CBD formulation, defined as the dose that achieves symptom relief with acceptable side-effects.
2. ESAS TSDS at days 7, 21 and 28.
3. Physical and emotional ESAS scores at each time point.
  Physical scores will be measured by The Australia modified Karnofsky Performance Status (AKPS) and Resource Utilisation Groups- Activities of Daily Living Scale (RUG-ADL)*.* The AKPS is a validated variant of the Karnofsky Performance Status. The Australian version can be applied to both in and outpatients and is sensitive to changes in function over time [14]. The RUG-ADL is an instrument developed for the measurement of nursing dependency. The ADL scale measures patients’ needs for assistance in activities of daily living (eating, bed mobility, transferring and toileting) [17].
4. Individual symptom scores (descriptive analysis only).
5. Oral morphine equivalent (OME), average use at baseline and weekly.
6. SLUMS score baseline and day 14.
  A person’s capacity to understand the study will be measured using the St Louis University Mental Status Examination. This tool is more sensitive than the Mini Mental Status Examination, and takes school education into account in the score. A score of 23 out of 30 will be considered an appropriate score to assess the ability to complete the study assessments [16].
7. Patient Global Impression of Change (PGIC), days 7 and 14, 21 and 28
  This is a subjective measure of symptom change done by the patient themselves [18].
8. Clinical Global Impressions (CGI) Scale, days 7 and 14, 21 and 28
  This is a subjective measure of symptom change done by the investigating clinician [18].
9. DASS-21 score baseline, days 14 and 28
  DASS-21 is a self-reported 21-item scale, 7 questions per sub-item questionnaire measuring depression, anxiety and stress. The DASS-21 will be used for assessment at baseline (day 0), days 7, 14 and 28 ^19^ EORTC score QoL baseline, days 14 and 28 [19, 20].
  This is a QoL measure found valid for use in a wide variety of cancer populations. To reduce patient burden, we will use the 15 question subset commonly used for the palliative care population.
10. NCI common terminology for adverse events V4.03, day 2, 4, 7, 9, 11, 14, 16, 18, 21, 23, 25 and 28 [21].
  The NCI CTCAE is a severity grading system for adverse events. Adverse events relate to an unfavourable or unintended sign, symptom or disease temporarily associated with a medical treatment or a procedure which may not be related to the medical treatment or procedure. The CTCAE has a grading of 1 (Mild) to 5 (Death). The known common adverse events associated with cannabinoids are: confusion, somnolence, paranoia, anxiety, mood changes, psychosis, hypertension, tachycardia, hyperhidrosis, nausea, vomiting and abdominal pain [21].

The study will assess adverse events (AE) and serious adverse events (SAE) using the criteria of the NCI, CTCAE V4 [21]. The known common AEs associated with CBD will be specifically addressed at each time point, including dry mouth, diarrhoea, light headedness and drowsiness, nausea, vomiting and abdominal pain). Finally, a detailed concurrent medication list is to be updated and recorded at baseline (day 0), days 7, 14, 21 and 28. This is to include over the counter medications, prescribed medications, complementary and antiemetic medication.

### Statistical analysis and sample size

Allowing 20% for attrition, and with improvement of ≥6 for CBD compared to placebo, it is anticipated that 144 participants (72 per arm) should be randomized to achieve a sample size of 60 participants per arm, assuming 80% power, a simple random sampling scheme and a Type 1 error of 5% (two-tailed), and a standard deviation of 11.6. The sample size is based on previous work by Hui et al. [13], who determined the minimal clinically important difference in the TSDS to be 5.7 [13]. As such we have elected to use an improvement of ≥6 in the TSDS as the primary outcome measure. The superiority of the CBD arm compared to placebo will be tested by comparing the response to each arm after 14 days, relative to baseline. Descriptive analyses and frequency distributions will be generated from participants’ demographic and clinical characteristics, with all variables explored using graphical methods and summary statistics. In univariate analysis, t-tests or the corresponding non-parametric tests (Wilcoxon Rank Sum) will be used to test for differences in change in total symptom distress scores of CBD versus placebo. Generalised estimating equation models with the appropriate link function will be developed to assess the effect of treatment and confounders and/or modifying factors on the primary and secondary outcomes and account for within subject correlation where required. This study is powered to detect superiority of CBD over placebo.

An interim analysis will be performed after 50% participants have completed 14 days of the trial. The analysis will be performed by a biostatistician blinded for the treatment allocation and reported to the investigators and the Data Safety Monitoring Board (DSMB). The purpose of the interim analysis is primarily to monitor and ensure safety of participants rather than evidence of such benefit that early stopping of the trial is justified. AEs and SAEs will be stratified by type and severity. The frequency of AEs and SAEs will be compared between treatment groups using chi-square test and logistic regression if indicated to adjust for any baseline differences between groups. If this shows a significant difference the investigators and DSMB will be un-blinded to the study groups and make any stopping decision on the basis of the nature of any AEs and/or SAEs and ethical grounds, as well as consideration of any statistical differences between the groups. Grounds for stopping on evidence of clear benefit will be considered.

### Data collection and management

Data will be sourced from a number of modes. The study is mainly based in the outpatient clinic, so most data will be collected from the participant and recoded in the corresponding CRF or questionnaires. Some data will be collected from medical records, but the majority of the data will be collected from:

**Table:** 

Measure	Source	Collected by
General medical information	Clinical record	Medical officer
General demographic data	Clinical record	Study nurse
Pathology results – blood	Pathology report	Pathology
Pathology results – urine	Clinical record	Study nurse
Vital signs	CRF/clinical record	Study nurse
Concurrent medications/OME	Clinical record	Study nurse
Study trial data - questionnaires – ASSIST, SLUMS, EORTC QLQ-C15-PAL	CRF	Patient, study nurse, medical officer `
Study trial data – ESAS, AKPS, RUG-ADL, PGIC, CGI-S/I, DASS – 21	CRF	Patient, study nurse, medical officer `
Side effects – safety	CRF, clinical record	Study nurse, medical officer

All data collected will be kept in a patient file (identified by ID number only). All data will be stored in a locked filing cabinet. At completion of the study, all CRFs will be collated and archived. Electronic files will be password protected and held within a locked office. All patient files will be reconciled and stored along with all study materials, both hard copy and electronic, consistent with the regulations of the hospital regarding the retention and disposal of patient records.

The trial will be conducted with permission and in accordance with Queensland Health regulations on the use of medical cannabis and subject to approval and monitoring by each clinical site’s HREC [4, 22]. An independent DSMB to include a statistician, clinical pharmacologist, palliative care specialist and consumers, will be formed and will meet regularly, with primary responsibility for monitoring adverse and serious adverse events. All AE’s and SAE’s will be reviewed at a minimum of 6 monthly intervals, or more frequently if needed.

## Discussion

The use of cannabis for symptom control continues to be a topical issue within medicine, and this study is the first placebo controlled, randomised trial to assess the efficacy of cannabinoids in advanced cancer patients. A major strength of this study is that it will target symptom burden as a whole, rather than individual symptoms, in an attempt to capture the improvement in general wellbeing reported anecdotally by many who have used cannabis [23, 24]. Randomisation with placebo is essential because of the well-documented over reporting of benefit in uncontrolled trials and high placebo response rates in cancer pain trials [25, 26]. The trial design is pragmatic, intended to allow as wide a range of participants as possible, to enable the results to be as real-world as possible. This study also allows for some data on dosing and formulation of CBD specifically.

## Data Availability

The datasets used and/or analysed during the current study are available from the corresponding author on reasonable request.

## Abbreviations

AE: Adverse Events
AKPS: Australia-modified Karnofsky Scale
ALT: Alanine Aminotransferase
ASSIST: Alcohol, Smoking and Substance Involvement Screening Test
AST: Aspartate Aminotransferase
BD: Twice Daily
CBD: Cannabidiol
CGI: Clinical Global Impressions scale
CINV: Chemotherapy induced nausea and vomiting
CPMP/ICH/135/95: Note for Guidance on Good Clinical Practice
CRF: Case Report Form
CRP: C-reactive protein
DASS – 21: The Depression, Anxiety, Stress Scale
DSMB: Data Safety Monitoring Board
EORTC QLQ-C15-PAL: European Organisation for Research and Treatment of Cancer
ESAS: Edmonton Symptom Assessment Scale
GCCS: Gold Coast Cancer Services
GD: Green Dispensary Pty Ltd. (GD Pharma)
GMP: Good Manufacturing Practice
HREC: Human Research Ethics Committee
ID: Identification
MANE: Morning
mg: Milligrams
mL: Millilitres
MML: Mater Misericordiae Limited
MRFF: Medical Research Future Fund
NHMRC: National Health and Medical Research Council
NOCTE: At night
OME: Oral Morphine Equivalent
QoL: Quality of Life
QPCRG: Queensland Palliative Care Research Group
RUG-ADL: Resource Utilisation Groups – Activities of Daily Living Scale
SAE: Serious Adverse Events
SLUMS: St Louis University Mental Status exam
TDS: Three times a day
TGA: Therapeutic Goods Administration
THC: Delta-9-Tetrahydrocannabinol
TSDS: Total Symptom Distress Score
